# The Impacts of Heating Strategy on Soil Moisture Estimation Using Actively Heated Fiber Optics

**DOI:** 10.3390/s17092102

**Published:** 2017-09-13

**Authors:** Jianzhi Dong, Rosa Agliata, Susan Steele-Dunne, Olivier Hoes, Thom Bogaard, Roberto Greco, Nick van de Giesen

**Affiliations:** 1 Water Resources Section, Faculty of Civil Engineering and Geosciences, Delft University of Technology, 2600 GA Delft, The Netherlands; jianzhi.dong@ars.usda.gov (J.D.); o.a.c.hoes@tudelft.nl (O.H.); t.a.bogaard@tudelft.nl (T.B.); n.c.vandegiesen@tudelft.nl (N.v.d.G.); 2 USDA ARS Hydrology and Remote Sensing Laboratory, Beltsville, MD 20705-2350 USA; 3 Dipartimento di Ingegneria Civile, Design, Edilizia e Ambiente, Università della Campania “L. Vanvitelli”, 81031 Aversa, Italy; rosa.agliata@unicampania.it (R.A.); roberto.greco@unicampania.it (R.G.)

**Keywords:** active DTS, soil moisture, soil temperature, heating strategy

## Abstract

Several recent studies have highlighted the potential of Actively Heated Fiber Optics (AHFO) for high resolution soil moisture mapping. In AHFO, the soil moisture can be calculated from the cumulative temperature ($T_{\mathrm{cum}}$), the maximum temperature ($T_{\max}$), or the soil thermal conductivity determined from the cooling phase after heating (λ). This study investigates the performance of the $T_{\mathrm{cum}}$, $T_{\max}$ and λ methods for different heating strategies, i.e., differences in the duration and input power of the applied heat pulse. The aim is to compare the three approaches and to determine which is best suited to field applications where the power supply is limited. Results show that increasing the input power of the heat pulses makes it easier to differentiate between dry and wet soil conditions, which leads to an improved accuracy. Results suggest that if the power supply is limited, the heating strength is insufficient for the λ method to yield accurate estimates. Generally, the $T_{\mathrm{cum}}$ and $T_{\max}$ methods have similar accuracy. If the input power is limited, increasing the heat pulse duration can improve the accuracy of the AHFO method for both of these techniques. In particular, extending the heating duration can significantly increase the sensitivity of $T_{\mathrm{cum}}$ to soil moisture. Hence, the $T_{\mathrm{cum}}$ method is recommended when the input power is limited. Finally, results also show that up to 50% of the cable temperature change during the heat pulse can be attributed to soil background temperature, i.e., soil temperature changed by the net solar radiation. A method is proposed to correct this background temperature change. Without correction, soil moisture information can be completely masked by the background temperature error.

## 1. Introduction

Soil moisture at point scales can be accurately measured using the gravimetric method and point-scale sensors, e.g., neutron probe, Time Domain Reflectometry (TDR), and heat-pulse sensors [1]. Upscaling these point sensor measurements to large scales is of great importance for hydrology and climate studies, but remains a significant challenge [2]. Several innovative techniques have been proposed to facilitate large scale soil moisture monitoring, e.g., cosmic-ray probes [3], GPS reflectometry [4,5] and distributed temperature sensing (DTS) e.g., [6,7]. Cosmic-ray probes and GPS reflectometry can only provide integrated soil moisture over large areas (approximately 700 m in diameter for cosmic-ray probe and 100 m for GPS reflectometry). Validating these techniques can be laborious and challenging due to the difficulty of upscaling point scale measurements. Several recent studies have demonstrated the feasibility of using DTS to provide cost-effective high-resolution soil moisture information over large areas [6,7,8,9,10,11]. DTS, therefore, has a potentially valuable role in bridging the observation gap from point-scale, to field-scale to satellite footprint scales. In addition to improving our understanding of soil moisture scaling, DTS may also be useful for combining or cross-validating measurements at different scales.

DTS is an advanced temperature measurement technique in which fiber optic cables are used to continuously map high resolution (temporal resolution <1 min, spatial resolution <1 m) soil temperature information [12]. In actively heated fiber optics (AHFO), the fiber optic cables are heated using electrically-generated heat pulses. Bulk soil thermal properties (specific heat capacity and thermal conductivity) are affected by soil water content. Therefore, the DTS measured temperature change after the heat pulse is a function of soil moisture content. Hence, AHFO has the potential to map soil moisture over large areas with high spatial and temporal resolutions. Sayde et al. [6] first demonstrated the potential of quantifying soil moisture using the AHFO. They installed DTS cables into a soil column, and used a relatively short (2 min) and strong (20 W/m) heat pulse to heat the DTS cables. They demonstrated that the cumulative cable temperature change ($T_{\mathrm{cum}}$) is a good indicator of soil moisture. In a subsequent study, Sayde et al. placed DTS cables at three depths (30, 60 and 90 cm) along a 240 m transect in an agricultural field in Oregon [13]. They showed that the $T_{\mathrm{cum}}$ collected using pulses of 10 W/m with 1 min duration could be used to detect the spatial variability of the soil moisture content. Gil-Rodriguez et al. also demonstrated that the $T_{\mathrm{cum}}$ method provided satisfactory estimates of the soil water distribution around drip emitters [14]. Striegl and Loheide II used longer (minimum of 10 min), lower power (3.07 W/m) heat pulses to estimate the soil moisture content at 20 cm depth along a transect of a floodplain of the Upper East Branch Pecatonica River. Instead of using $T_{\mathrm{cum}}$, the maximum temperature increase after heating ($T_{\max}$) was used to estimate soil moisture [15]. They demonstrated that this $T_{\max}$ performed well, particularly under relatively dry conditions. Ciocca et al. proposed a method to estimate soil thermal conductivity using the cooling process of the cable temperatures after heating [16]. Soil moisture can then be derived from the estimated soil thermal conductivity. To test the proposed method, they installed about 32 m of fiber optic cables in a lysimeter. Heat pulses with duration of 2 min, and strengths of 11 and 36 W/m were used. The reported error (0.01 to 0.035 $m^{3}/m^{3}$) is similar to that reported for the $T_{\mathrm{cum}}$ and $T_{\max}$ methods.

The three heat pulse analysis methods exploit different features of the soil thermal responses, e.g., the total undissipated energy for $T_{\mathrm{cum}}$, maximum temperature increase for $T_{\max}$ and the cooling process for the λ method. These features are determined by the choice of heating strategies. Hence, the comparative performance of the methods may depend on the heating strategy adopted, e.g., heating duration and heating strength. For a given soil type and soil moisture condition, different heating strategies lead to different heating and cooling rates and maximum temperature increase. This is particularly relevant to improve the AHFO performance for soil moisture monitoring in field conditions, since the duration and/or strength of heat pulses that can be generated in the field might be limited by the available power supply. For example, when only portable electric generators are available to supply power in the field, the maximum output power is typically <5 kW. Hence, the maximum strength of the heat pulses will be less than 5 W/m to heat 1 km fiber optic cables. Therefore, it is important to know which of the three heat pulse analysis methods will yield the best performance given this constraint. More importantly, the heating strategies (i.e., heating duration and strength) employed in previous studies varied widely, making inter-comparison difficult. The study presented here is the first time that the performance of the three approaches has been compared for different heating strategies.

The key objective of this study is to explore the impact of different heating strategies on the three heat pulse analysis methods. In particular, we want to know which (if any) approach performs best in a situation where the power supply is limited to a portable electric generator. This is important if AHFO is to be considered as a viable, and feasible tool for soil moisture monitoring. To do this, we analyzed the three heat pulse processing methods using three different heating strategies across the full range of moisture values of a sand. Finally, we also investigated the influence of the soil background temperature change on the estimated soil moisture from AHFO.

## 2. Materials and Methods

### 2.1. Experimental Site and Data Collection

**Figure 1:**
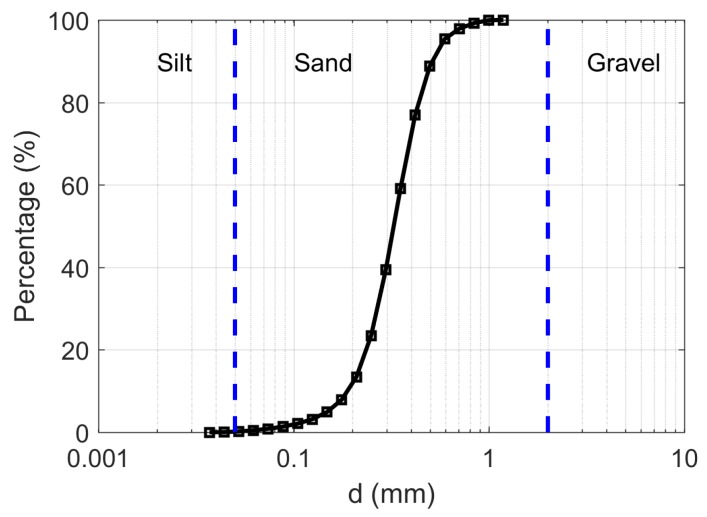
Particle size distribution of the sand used for testing AHFO soil moisture estimation.

**Figure 2:**
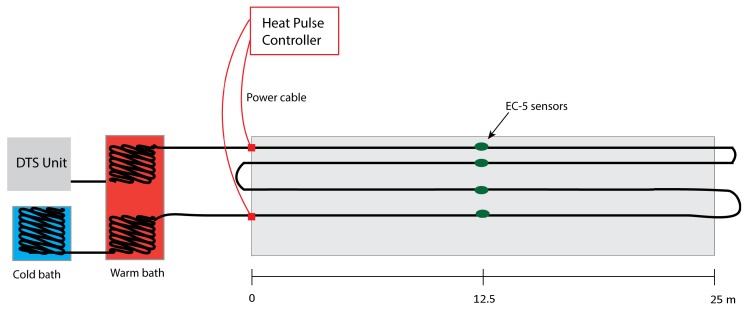
An illustrative diagram of the experiment setup.

Data were collected at an experimental set-up near Delft in the Netherlands (51.98${}^{\circ }$ N, 4.38${}^{\circ }$ E). A concrete container 25 m long, 3 m wide and 0.4 m deep was built in July, 2014. The container was lined with EPDM waterproofing foil, and a perforated PVC pipe was laid in a layer of gravel to ensure free drainage. The container was filled by adding four 0.1 m deep layers of sand. After each layer was added, the sand was gently packed, its surface was smoothed with a wooden beam, and water was added to near-saturation. This ensured a relatively homogeneous vertical distribution of bulk density. The dry bulk density of the sand was determined (1.53 $g/{\mathrm{cm}}^{3}$) and the measured particle size distribution is shown in Figure 1. At the time of this experiment, the surface was bare with sparse weeds with a maximum root depth of 5 cm. Armored two-fiber multimode (50/125 μm) optic cable from Kaiphone Technology (Dongguan, China) was laid in a loop to observe soil temperature at depths of 2.5, 5, 10 and 20 cm (Figure 2). The soil temperature data were collected using a Silixa Ultima-S (Silixa Ltd., Hertfordshire, UK), with a spatial resolution of 0.29 m, and temporal resolution of 5 s. The Ultima-S unit used in this study is a standalone unit, with an on-board PC. The DTS unit is available with 4 channels, which can be configured for both single-ended and double-ended soil temperature measurements. The DTS temperature data were collected using single-ended configuration and calibrated using the method presented in [17]. The first heat pulse applied (H5) used a power input of 9.2 W/m with a duration of 5 min. This corresponds to the the highest possible input energy without overheating the exposed DTS cables. In the second case (L5), the power was then lowered by half and the same heating duration (5 min) was used. Finally, a low power input (4.6 W/m) was combined with a longer duration (10 min), denoted as L10. A comparison of the characteristics of the three heating strategies is shown in Table 1. These three heat pulses strategies were employed to measure on several days between 3 and 19 June 2016. The timing of data collection was primarily determined by the weather conditions, so the data were collected at irregularly spaced time intervals in this period.

Soil moisture validation data were collected using four EC5 soil moisture sensors (Decagon Devices, Pullman, WA, USA) installed at the same depths as the DTS cables, i.e., 2.5, 5, 10 and 20 cm, and approximately 5 cm away from the DTS cables (Figure 2). The (0.29 m) section of the DTS cable closest to the EC5 sensor was located using ice bags at each depth. The EC5 sensors were calibrated using the gravimetric method on cores of the sand used to fill in the concrete container. The error of the calibrated EC5 sensors ranged between 0.01 and 0.02 $m^{3}/m^{3}$, compared with the gravimetric measurements. Since the aim of this study is to investigate the impacts of heating strategies on the AHFO method, rather than the spatial variability of the soil moisture, only one soil moisture profile was observed with the EC5 sensors for validating the AHFO method. Two rain simulators, located at approximately 7.5 m and 32.5 m along the container, were used to apply artificial rain and to achieve different water contents. The water used for the rain was pumped from a canal located 10 m away from the container. Soil moisture was measured every 10 min using the EC5 sensors. AHFO measurements were conducted when the water content stopped changing (i.e., differences <0.01 $m^{3}/m^{3}$). Soil thermal conductivity of the sand as a function of soil moisture content (i.e., soil thermal conductivity curve) was also measured using a dual-probe heat-pulse sensor (KD2Pro, Decagon Devices).

### 2.2. Soil Moisture Estimation Methods

#### 2.2.1. The $T_{\mathrm{cum}}$ Method

The cumulative temperature increase after heating, $T_{\mathrm{cum}}$, is calculated as follows [6]:

(1) $$T_{\mathrm{cum}}=\int_{t_{s}}^{t_{e}}\Delta T\left(t\right)dt,$$

where ΔT is the temperature increment with respect to the background temperature (K), $t_{s}$ and $t_{e}$ are the time of start and the end of the heat pulse, respectively (s). The $T_{\mathrm{cum}}$ can be related to soil moisture using empirically calibrated equations.

#### 2.2.2. The $T_{\max}$ Method

The $T_{\max}$ is usually calculated as an average of temperature data collected over a certain period of time once the temperature rise has plateaued [15]:

(2) $$T_{\max}=\frac{1}{N}\sum_{t_{e}-\Delta t}^{t_{e}}\Delta T\left(t_{i}\right),$$

where Δt is the length of the period (s) used for calculating $T_{\max}$, which is set to be 120 s in this study, and *N* is the number of the measurements within this period.

#### 2.2.3. The λ Method

In the λ method [16], the cooling phase of the heat pulse is used to estimate the soil thermal conductivity (λ) by fitting the following equation:

(3) $$\Delta T\left(t\right)=\frac{Q}{4\pi \lambda }ln\left(\frac{t+t_{0}}{t-\Delta t_{h}+t_{0}}\right),$$

where *Q* is the strength per unit cable length of the constant source of heat (W/m), $\Delta t_{h}$ is duration of the heat pulse, and $t_{0}$ is a time correction term, which is estimated together with λ.

As noted above, the thermal conductivity (λ) is estimated by fitting Equation (3) to the cooling phase of the heat pulse. In [16], the start of the cooling phase is considered as 90 s (threshold time) after heating. However, due to the low temperature increase in this study, a threshold time of 10 s provides the most reasonable estimates.

### 2.3. Background Temperature Correction

**Figure 3:**
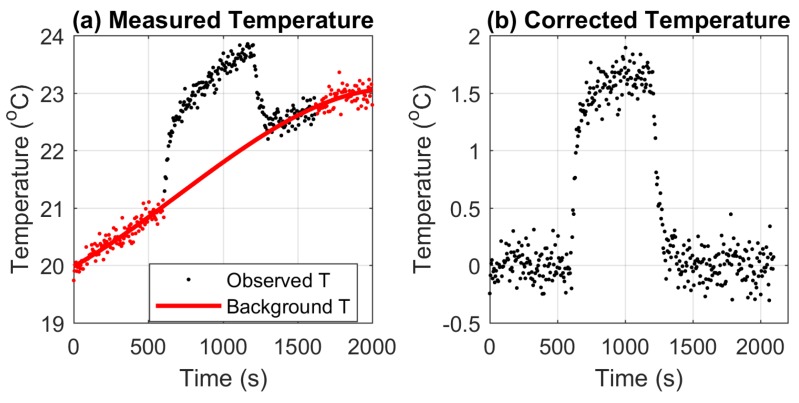
Soil temperature measurement during a heat pulse before (**a**) and after (**b**) background temperature correction. Red dots in (**a**) are the temperature measurements used for estimating the background temperature (red solid line) during the heat pulse.

The background temperature is estimated by fitting the temperature measurements before and after the heat pulses using a third order polynomial equation. Other equations may also be used for fitting the background temperature, but a third order polynomial was found to be flexible enough to provide accurate estimates at different stages of the daily net radiation cycle. As shown by [16], the measured temperature can cool back to the background temperature after 300 s (5 min) of cooling, even when the increase in temperature is as high as 30 ${}^{\circ }$C. Hence, soil temperatures from the 5 min prior to the start of the heat pulse, and temperatures 5 to 10 min after the heat pulse are combined to estimate the background temperature (red line in Figure 3a). This estimated background temperature is removed from the measured soil temperature to obtain the corrected cable temperature (Figure 3b).

### 2.4. AHFO Evaluation Metrics

The AHFO estimated soil moisture will be validated against the EC5 soil moisture measurements using two metrics. The first is the root mean squared difference (RMSD) given by:

(4) $$RMSD=\sqrt{\frac{1}{n}\sum_{i=1}^{n}{\left(\theta_{est,i}-\theta_{obs,i}\right)}^{2}},$$

where $\theta_{est}$ and $\theta_{obs}$ represent the AHFO estimated and the EC5 observed soil moisture, respectively, and *n* is the number of the data points. Note that the AHFO method is also calibrated using the EC5 sensors. Hence, the reported RMSD of the AHFO method essentially measures the differences between the AHFO and the EC5 in the validation phase, rather than the absolute error of the AHFO method.

A second metric used for validating the AHFO method is the coefficient of determination ($R^{2}$). It represents the goodness of fit of the data to the regression curve:

(5) $$R^{2}=1-\frac{\sum_{i=1}^{n}{\left(y_{i}-f_{i}\right)}^{2}}{\sum_{i=1}^{n}{\left(y_{i}-\bar{y}\right)}^{2}},$$

where $y_{i}$ is the measured value and $f_{i}$ is the corresponding estimated value. $R^{2}=1$ means the estimated values fits the observations perfectly. Negative values of $R^{2}$ (i.e., $\sum_{i=1}^{n}{\left(y_{i}-f_{i}\right)}^{2}<\sum_{i=1}^{n}{\left(y_{i}-\bar{y}\right)}^{2}$) indicate that the predictions provide little or no information about the observations.

## 3. Results and Discussion

### 3.1. Comparison of the Heat Pulse Analysis Methods

**Figure 4:**
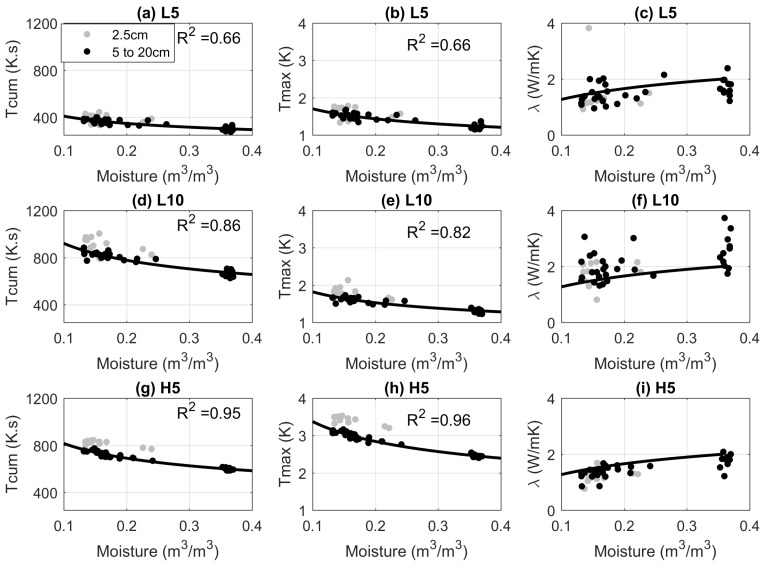
$T_{\mathrm{cum}}$ (first column), $T_{\max}$ (second column), and the estimated soil thermal conductivity (λ) (third column) as a function of EC5 measured soil moisture. The solid lines in the first two columns, are the fitted $T_{\mathrm{cum}}$ and $T_{\max}$ to soil moisture relationship, in which 2.5 cm measurements were not considered. The solid lines in the third column represents the measured soil thermal conductivity curve using KD2Pro heat-pulse sensor. Each plot represents heat pulse and soil moisture data collected from all four depths.

Figure 4 shows how the measured $T_{\mathrm{cum}}$ and $T_{\max}$, as well as estimated λ vary with soil moisture for each of the heating strategies. Each row corresponds to a heating strategy and each column to a soil moisture estimation method. The estimated values of λ are compared to those estimated from a λ(θ) relationship determined from the independent measurements of soil thermal conductivity.

When short pulses with low power input (L5) are used, the magnitudes of $T_{\mathrm{cum}}$ and $T_{\max}$ are low, and the dynamic range of values is limited (Figure 4a,b). For this heating strategy, the estimated λ appears to be a weak function of soil moisture. Consequently, this method sometimes returns physically implausible estimates of λ (values larger than 3 W/mK, Figure 4c). Increasing the length of the pulse (i.e., using L10) causes a modest increase in both the magnitude and range of $T_{\max}$ values obtained. It has a more significant effect on $T_{\mathrm{cum}}$, which, in this case, yields marginally better results than the $T_{\max}$ approach (Figure 4d,e). Extending the pulse duration has a negative effect on the λ method. The results are very scattered, and it is impossible to identify a clear relationship between λ and the water content (Figure 4f).

Using a shorter pulse but with higher input power (H5) leads to a significant increase in the $T_{\max}$ values obtained compared to L5. The $T_{\mathrm{cum}}$ and $T_{\max}$ methods yield comparable results in this case (Figure 4g,h). The higher power input also benefits the λ approach. The results are less scattered than the L5 case, producing a better-defined relationship between the estimated λ and soil moisture. The estimated relationship is also close to the independently derived moisture—λ(θ) relationship (Figure 4i). This is because starting the cooling phase at a higher temperature reduces the relative impact of measurement noise. Hence, the observations provide a better fit to Equation (3).

**Figure 5:**
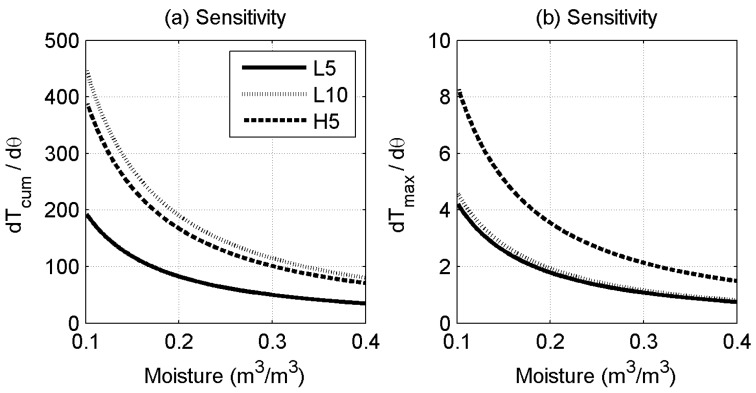
The sensitivity $T_{\mathrm{cum}}$ (**a**) and the $T_{\max}$ (**b**) to soil moisture when different heating strategies were used. The sensitivity curves are derived from the fitted $T_{\mathrm{cum}}$ and the $T_{\max}$ to soil moisture relationship in Figure 4.

Figure 5 shows the influence of the heating strategy on the sensitivity of $T_{\mathrm{cum}}$ (a) and $T_{\max}$ (b) to soil moisture. Since $T_{\mathrm{cum}}$ essentially measures the total undissipated energy, similar sensitivity can be achieved using the lower input power for a longer duration or using the higher input power for the shorter pulse for this approach. Figure 5b shows that for the $T_{\max}$ approach, extending the heat pulse duration has no effect, while increasing the input power leads to increased sensitivity. Hence, if input power is the limiting constraint, the $T_{\mathrm{cum}}$ approach is preferable. However, it is also worth noting that, for the same (higher) power input, the $T_{\max}$ yields marginally better results.

In Figure 4, it is noteworthy that the relationships linking soil moisture with $T_{\mathrm{cum}}$ and $T_{\max}$ at 2.5 cm are significantly different from those at higher depths, e.g., the $T_{\mathrm{cum}}$ and the $T_{\max}$ measured at 2.5 cm are consistently larger than those measured in deeper layers at the same soil moisture conditions. The proximity of the EC5 sensors to the soil surface explains some of this bias. First, offline testing of the sensors in a laboratory experiment with the same sand confirmed that the measurement volume of the sensor at 2.5 cm depth includes air. Second, the soil moisture values were also sensitive to the orientation of the EC5 sensor at this depth. The measurement volume of AHFO is a topic of ongoing research. Thus, there is no reason to assume that AHFO and EC5s are measuring the same sampling volume. Therefore, sharp thermal and moisture gradients close to the surface [18,19], combined with the difference in measurement volume between the AHFO and EC5 result in a mismatch between the soil moisture being observed by the two methods. Another possible cause of mismatch is that macro-pores in the upper soil, due to the presence of roots, could influence the porosity and moisture transport near the sensors, and the presence of air gaps around the cables could affect the temperature observations of the AHFO.

### 3.2. Comparison of the Estimated Soil Moisture

**Figure 6:**
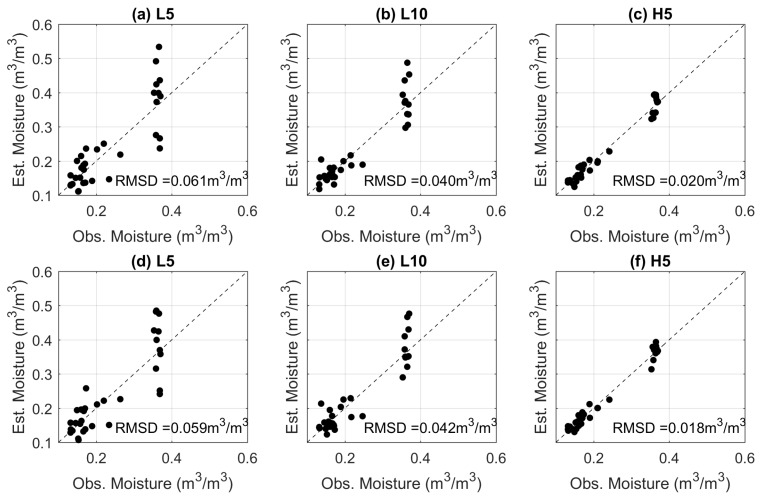
Comparison of the observed and the estimated soil moisture using the $T_{\mathrm{cum}}$ method (**a**–**c**) and $T_{\max}$ method (**d**–**f**). Soil moisture measurements at 2.5 cm were not included.

The empirical fits applied to the data in Figure 4 were used to estimate soil moisture from the AHFO measurements. As the estimates from the λ method regularly exceed the physically reasonable range (Figure 4, third column), only the $T_{\mathrm{cum}}$ and $T_{\max}$ methods are considered here. The results are compared to the soil moisture values from the EC5 sensors in Figure 6. As expected, L5 gives the poorest results for both methods and increasing the heating duration (L10) leads to a 50% reduction in RMSD. It is noteworthy that when the power input is increased (H5), the estimated soil moisture RMSD is around 0.018 to 0.02 $m^{3}/m^{3}$, which is comparable to the soil moisture sensor measurement error.

The spread around the 1:1 lines indicates that the soil moisture error increases with soil wetness, which is consistent with previous studies [6,15,16]. This is particularly noticeable when the lowest power input is used. In Figure 6a, when the observed soil moisture from the EC5 is around 0.35 $m^{3}/m^{3}$, the estimates from the AHFO methods vary between 0.2 to 0.5 $m^{3}/m^{3}$. When a higher input power is used, the spread of estimated moisture values from both approaches is dramatically reduced (Figure 6c). Thus, increasing the power input is particularly effective in reducing the errors at high moisture content.

### 3.3. The Impact of Background Temperature Correction

**Figure 7:**
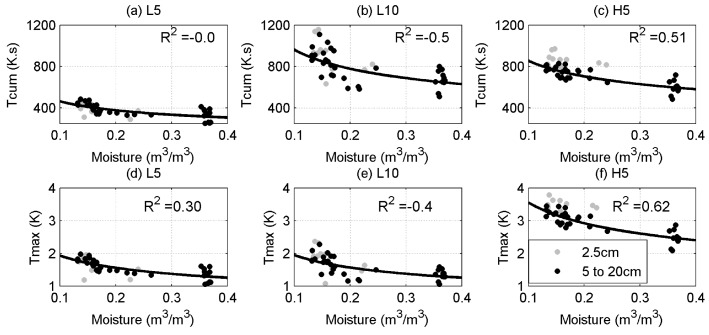
Same as Figure 4 but without background temperature correction.

In the results presented so far, the background temperature had been estimated and removed from the thermal response to the heat pulse as described in Section 2.3. Figure 7 shows the relationship of $T_{\mathrm{cum}}$ and $T_{\max}$ as a function of soil moisture in the case where background temperature is not corrected. Note that a small or even negative $R^{2}$ indicates that background temperature error completely dominates the soil moisture information in the heat pulses. Compared to Figure 4, it is clear that the background temperature correction results in a significant increase in $R^{2}$ in both the $T_{\mathrm{cum}}$ and $T_{\max}$ methods. This improvement is substantial, particularly taking into account that the daily soil temperature amplitude at 2.5 cm during this experiment was less than 5 ${}^{\circ }$C. This effect may not have been considered in previous studies because the power input was high enough that the change in background temperature was small compared to the heat pulse. Furthermore, the experiments were conducted in laboratory, under controlled ambient conditions [6], and/or the cables were deep enough that the temperature did not vary considerably during the day [13,15,16]. One of the potential applications for AHFO is the validation of soil moisture estimates from remote sensing, an application which would require cables not deeper than 5 cm from the surface. At locations with higher solar radiation than the Netherlands, it is expected that the soil background temperature correction would prove to be even more important.

## 4. Conclusions

This study investigated the impact of heating strategy on the performance of three approaches commonly used to estimate soil moisture using AHFO: the cumulative temperature ($T_{\mathrm{cum}}$), the maximum temperature ($T_{\max}$), and the soil thermal conductivity (λ) determined from the cooling phase after heating. Results show that increased power input improves the accuracy of all three AHFO methods. For the λ method, increasing the pulse length has no influence, but the method fails if the power input is not large enough. If the available power input is limited, one of the temperature based (i.e., $T_{\mathrm{cum}}$ and $T_{\max}$) methods should be employed instead. For the $T_{\mathrm{cum}}$ method, increasing the heat pulse duration can produce the similar improvement in sensitivity as an increase in power. However, increasing power is more effective in reducing RMSD than increasing the pulse length. This is particularly true for wet conditions. Though the $T_{\mathrm{cum}}$ method has better sensitivity to soil moisture, the RMSD and $R^{2}$ values from both empirical temperature-based methods are similar.

It was demonstrated that variations in background temperature can have a significant influence on the quality of the estimated soil moisture. This becomes particularly important in field applications where the heat increase due to the pulse is small compared to the dynamics of background temperature. Estimating the background temperature change by assuming a third order polynomial equation and removing it prior to estimation significantly improved the accuracy of AHFO approaches even when the background variations were quite small. This correction is recommended for applications in which AHFO cables are close enough to the surface to be affected by the daily temperature cycle.

Since the main scope of this study is to investigate the impacts of heating strategies on AHFO, the soil texture (sand) remained unchanged. Some differences are to be expected when soils with a higher clay content are employed. However, the results and conclusions with respect to heat strategies are unlikely to be influenced by soil type. Physically based numerical modelling of the heat diffusion in and around the fiber-optic cables is recommended to further investigate the effects of heating strategies on the AHFO estimates, and the impacts of the soil thermal properties on the AHFO methods.

## Figures and Tables

**Table 1:** The strength and the duration of the pulse in the three heating strategies.

Strategy	L5	L10	H5
Strength (W/m)	4.6	4.6	9.2
Duration (min)	5	10	5
